# Empirical Evaluation of Bone Extraction Protocols

**DOI:** 10.1371/journal.pone.0031443

**Published:** 2012-02-14

**Authors:** Timothy P. Cleland, Kristyn Voegele, Mary H. Schweitzer

**Affiliations:** 1 Department of Marine, Earth, Atmospheric Sciences, North Carolina State University, Raleigh, North Carolina, United States of America; 2 Biology Department, Concordia College, Moorhead, Minnesota, United States of America; 3 North Carolina Museum of Natural Sciences, Raleigh, North Carolina, United States of America; Monash University, Australia

## Abstract

The application of high-resolution analytical techniques to characterize ancient bone proteins requires clean, efficient extraction to obtain high quality data. Here, we evaluated many different protocols from the literature on ostrich cortical bone and moa cortical bone to evaluate their yield and relative purity using the identification of antibody-antigen complexes on enzyme-linked immunosorbent assay and gel electrophoresis. Moa bone provided an ancient comparison for the effectiveness of bone extraction protocols tested on ostrich bone. For the immunological part of this study, we focused on collagen I, osteocalcin, and hemoglobin because collagen and osteocalcin are the most abundant proteins in the mineralized extracellular matrix and hemoglobin is common in the vasculature. Most of these procedures demineralize the bone first, and then the remaining organics are chemically extracted. We found that the use of hydrochloric acid, rather than ethylenediaminetetraacetic acid, for demineralization resulted in the cleanest extractions because the acid was easily removed. In contrast, the use of ethylenediaminetetraacetic acid resulted in smearing upon electrophoretic separation, possibly indicating these samples were not as pure. The denaturing agents sodium dodecyl sulfate, urea, and guanidine HCl have been used extensively for the solubilization of proteins in non-biomineralized tissue, but only the latter has been used on bone. We show that all three denaturing agents are effective for extracting bone proteins. One additional method tested uses ammonium bicarbonate as a solubilizing buffer that is more appropriate for post-extraction analyses (e.g., proteomics) by removing the need for desalting. We found that both guanidine HCl and ammonium bicarbonate were effective for extracting many bone proteins, resulting in similar electrophoretic patterns. With the increasing use of proteomics, a new generation of scientists are now interested in the study of proteins from not only extant bone but also from ancient bone.

## Introduction

The application of non-traditional, high-resolution analytical techniques to the study of ancient bone proteins holds great potential for increasing our understanding of the evolution, radiation, and ecology of extinct organisms. However, these analyses are challenging because they require the detection and interpretation of molecular components that are present at very low concentration and/or are highly altered. All of these high-resolution techniques require some form of protein extraction, and bone provides unique challenges for extraction of its protein components. Unlike soft tissues, proteins present in bone are secreted by osteoblasts and subsequently biomineralized with hydroxyapatite (Ca_5_(PO_4_)_3_OH; [1], [2]). It has been proposed that the presence of these minerals, in addition to providing stability to collagen I [3], [4], can prevent enzymatic digestion, because the enzymes are simply too large to ‘fit’ between the crystals to access the protein [3], [5], [6], [7]. This biomineralization has been proposed to contribute to the preservation of collagen I through geological time [5]. Thus, this intimate association with mineral affords protection from degradation not found in non-mineralized tissues [3], [7]. Additionally, the presence of minerals on which biomolecules may adsorb provides stabilization to both the molecules and to the mineral [3], [6].

These challenges have led investigators to propose many different protocols (>20 variants on 3–5 methods) for demineralization and extraction of bone for protein analyses. Because of the many variants, choosing the best method for a protein of interest or particular analytical technique becomes challenging. Generally, investigations of bone protein use a weak inorganic acid [8], [9], a diluted strong acid [10], [11], [12], [13], [14], or ethylenediaminetetraacetic acid (EDTA; [14], [15], [16], [17], [18], [19], [20], [21], [22], [23], [24], [25]) for demineralization. However, the use of either weak inorganic acids or diluted strong acids may hydrolyze the proteins of interest, and therefore, cleave them into difficult to characterize fragments [26], [27]. EDTA ligates calcium removing it from the mineral lattice. By removing the calcium from the mineral, it releases the phosphate into solution resulting in demineralization.

After mineral has been removed, bone proteins are typically extracted into solution for further analyses. In non-biomineralized tissues, this is usually accomplished by urea [28], [29] or sodium dodecyl sulfate (SDS; [30], [31]), but these methods have seldom been used in bone. These methods create challenges because they can modify proteins by forming adducts (SDS; [32]) or by carbamylation (urea; [29]). Instead, guanidine HCl [8], [13], [15], [16], [17], [18], [19], [20], [21], [24], [25] or ammonium bicarbonate [10], [11], [12] have been found to effectively solubilize bone proteins. Guanidine HCl functions by denaturing proteins into random coils, making them more soluble [31], but many proteins are soluble in ammonium bicarbonate as well. Ammonium bicarbonate is widely used in proteomics-based techniques because it completely breaks down to ammonia and carbon dioxide and is directly compatible with typical digestive enzymes (e.g., trypsin) without the need for desalting [9], [10], [11].

Once the mineral has been removed and proteins extracted, previous investigators have characterized these proteins using molecular biological techniques [19], [20], [21], [33], mass spectrometry [10], [11], [21], [34], amino acid analysis [11], [35], [36], [37], and/or assessment of stable isotopes [14], [38], [39]. Each technique requires different concentrations of the proteins of interest, different sample preparation, and differing degrees of sample purity. For example, molecular methods utilizing antibodies can detect very small concentrations of a protein, either in situ or in solution, based on epitope identification [7], [40]; however, these techniques do not provide primary sequence information, and therefore, only allow for crude phylogenetic inference [40], [41], [42], [43], [44], [45], [46], [47]. Mass spectrometry, on the other hand, requires greater concentrations of proteins and can provide primary sequence, but high concentration of salts can interfere with ionization and interpretation [48]. Amino acid analysis has been used extensively for racemization studies to determine kinetics of amino acid changes within bone collagen, crudely determining protein content, and assessing the amount of protein degradation (e.g., [11], [35], [36], [49], [50]); however, this method results in the loss of primary sequence information and cannot address all amino acids from a sample because some amino acids are not stable under necessary acidic hydrolysis conditions [51]. Stable isotope studies also do not provide primary sequence information, but can provide additional environmental and ecological information for the organisms studied (e.g., [38], [39], [52]).

Bone is composed of a variety of proteins and other molecules, but archaeologists and paleontologists have focused on collagen I (e.g., [10], [11], [19], [20], [21], [34], [53], [54]) and osteocalcin (e.g., [55], [56], [57], [58]) because they are the two most abundant proteins in extant bone and both have high potential for preservation [6]. Collagen I is, by far, the most dominant protein, making up ∼85–90% of the organic constituents in bone [2]. Because collagen is vital for bone structure and formation, its sequence is highly conserved across taxa, making it less useful for determining relationships of extinct organisms [41]. It has, however, been used to determine vertebrate relationships at the species [21], [59] and supraspecific level (e.g., being able to identify bone from elephantidae but not from a specific taxon; [53]). Non-collagenous proteins (NCPs) have sequences with greater sequence variation providing potentially greater phylogenetic resolution than collagen I. However, identifying these proteins in bone extracts is difficult, even in extant bone, because they make up a relatively small fraction of the total protein content. If these proteins are not preferentially collected or concentrated, their signal can be overwhelmed by much more abundant proteins. Recently, investigators have used mass spectrometry to study NCPs [8], [9], [13], which may be used to provide a better understanding of relationships between extinct organisms, and in addition may elucidate diagenetic processes present within bone.

Here, we compare previously described methodology for demineralizing and extracting proteins from bone. We use extant ostrich (*Struthio camelus*) bone as a baseline for the expected protein composition of bone, and extinct moa bone as an exemplar for what is expected for ancient material. Both electrophoretic separation and enzyme-linked immunosorbent assay (ELISA) are employed to compare these different methods for yield and purity.

## Results and Discussion

### Modern Bone Protein Extraction Methods

**Figure 1:**
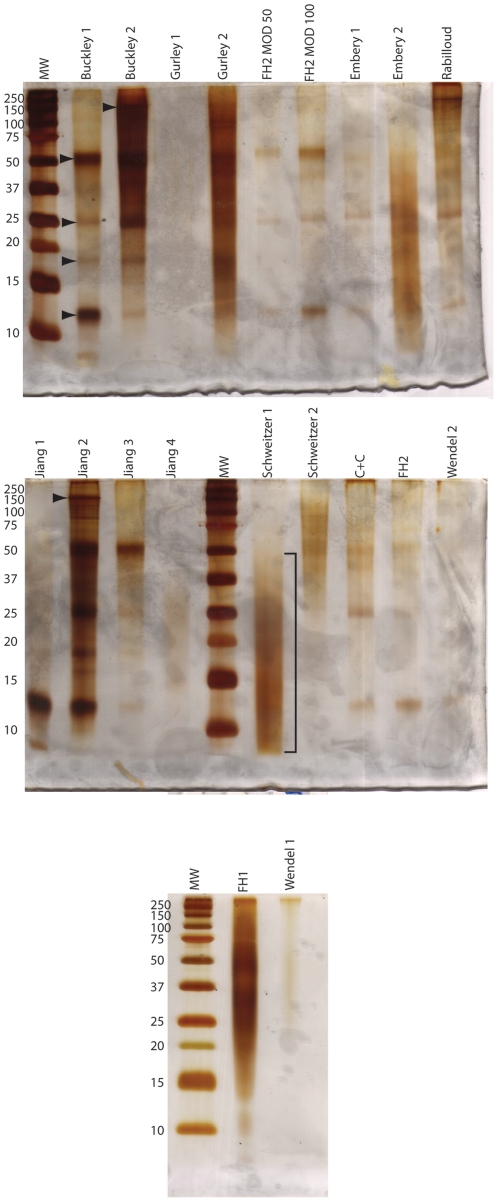
Sodium dodecyl sulfate polyacrylamide (SDS-PAGE) gels of ostrich extractions. Arrowheads indicate bands observed in many different extractions. The bands at ∼12 kDa likely correspond to osteocalcin, which can act as a dimer under these conditions. The bracket next to the Schweitzer 1 lane indicates smearing with no apparent banding.

Even though many different protocols (Tables 1, 2, 3, 4) have been used for preparing ostrich bone samples, they all show some amount of protein solubilization, resulting in a white powder after lyophilization, with yields ranging from negligible to 29.5% (Table 5). Urea/thiourea and SDS have seldom been used for solubilization of protein in bone, but they have both been shown to be effective in this study on both gels and in ELISA. Both methods show at least some antibody binding in ELISA (C+C and Rabilloud, Table 6) and a few bands on gels (Fig. 1). SDS has additional post-extraction benefits including usage in polyacrylamide analyses (e.g., in gel digestion for mass spectrometry, Western blotting) without additional sample cleanup. It can, however, impact mass spectrometry by forming adducts to proteins/peptides making characterization more difficult [32]. Like SDS, urea can be used in polyacrylamide gel electrophoresis without removal [29], [60] and is especially good for low molecular weight proteins [60]. Buffers containing urea can also be used directly for mass spectrometry (e.g., [61]), but care must be taken not to overheat the samples leading to carbamylation [29]. Urea does not precipitate in the presence of SDS, unlike guanidine HCl [62], so less desalting is necessary for urea extractions than for guanidine HCl ones. However, we found that neither SDS or urea was as effective as methods utilizing guanidine HCl or ammonium bicarbonate based on ELISA and gel data, but they still could be used for bone. Further methodological development is necessary to evaluate benefits of SDS or urea in characterization of the bone proteome, especially with the usage of HCl for demineralization. The ammonium bicarbonate extraction (Buckley 2) and guanidine HCl extraction (Jiang 2) following HCl demineralization show very few differences. Both methods show collagen I detection by ELISA (Table 6) and very little difference on gel (Fig. 1). The Jiang method results in a smaller yield than the Buckley method for approximately the same starting amount of bone, so if greater yield is necessary, the Buckley method may be better. The yield in the Jiang steps is very small because the overall volume is small per step, but enlarging the volume may help overcome protein saturation levels for the solvents. Larger volumes give larger yields based on comparable methods and their yield percentages. For example, the Schweitzer method uses a total of four volumes of buffer per step and the Franzén and Heinegård method uses ten volumes of buffer per step. The Franzén and Heinegård method has over 17 times more yield than the Schweitzer method (Table 5). These large yields likely include residual salt, however, because the yields are greater than the protein content in bone (∼20%; [63]). The increase in yield allows for multiple assays to be performed without being material limited resulting in additional extraction periods. Future optimization of buffer capacity and volume should allow for high yield.

**Table 1:** Summary of Franzén and Heinegård methods.

Citation	Sample Name	Solution	Purpose	Volume	Incubation Time	Sample treatment*
[16], [17]	FH1	Chilled 4 M GuHCl	Remove free protein	10 volumes	6 hr	D/L or precip
	FH2	250 mM disodium EDTA in 4 M GuHCl in 50 mM Tris pH 7.4	Demineralize and extract protein	3×10 volumes	3×24 hr	D/L or precip
Modified [16], [17]	FH1 6 M 50 mM	Chilled 4 M GuHCl	Remove free protein	10 volumes	6 hr	D/L
	FH2 6 M 50 mM	250 mM disodium EDTA in 6 M GuHCl in 50 mM Tris pH 7.4	Demineralize and extract protein	3×10 volumes	3×24 hr	D/L
	FH1 6 M 100 mM	Chilled 4 M GuHCl	Remove free protein	10 volumes	6 hr	D/L
	FH2 6 M 100 mM	250 mM disodium EDTA in 6 M GuHCl in 100 mM Tris pH 7.4	Demineralize and extract protein	3×10 volumes	3×24 hr	D/L

*Dialysis and lyophilization is abbreviated D/L. Precip represents chloroform∶methanol∶water precipitation performed on half of the supernatant. Volumes correspond to the number of milliliters of buffer multiplied by the grams of bone powder.

**Table 2:** Summary of Gurley method and Wendel method.

Citation	Sample Name	Solution	Purpose	Volume	Incubation Time	Sample treatment*	Additional notes
[18]		2 M HCl	Demineralize	20 volumes	48 hr	D/L	Neutralized with NaOH, lyophilized and extracted in Gurley 1
	Gurley 1	6 M GuHCl, 0.2% trifluoroacetic acid, 0.025% dithiothreitol, 0.155 M NaCl, 0.026 M HCl	Extract protein	3×6.5 mL	3×1 hr	D/L	On neutralized salt powder
	Gurley 2	6 M GuHCl, 0.2% trifluoroacetic acid, 0.025% dithiothreitol, 0.155 M NaCl, 0.026 M HCl	Extract protein	3×6.5 mL	3×1 hr	D/L	On pellet
[25]	Wendel 1	Chilled 4 M GuHCl	Remove free protein	10 volumes	6 hr	D/L or precip	
	Wendel 2	500 mM disodium EDTA in 4 M GuHCl in 50 mM Tris pH 7.4	Demineralize and extract protein	3×10 volumes	3×24 hr	D/L or precip	White precipitate formed during incubation. This was resolubilized at 60°C for 1–2 hr

*Dialysis and lyophilization is abbreviated D/L. Precip represents chloroform∶methanol∶water precipitation performed on half of the supernatant. Volumes correspond to the number of milliliters of buffer multiplied by the grams of bone powder.

**Table 3:** Summary of Rabilloud method, Craig and Collins method, Embery method, and Schweitzer method.

Citation	Sample Name	Solution	Purpose	Volume	Incubation Time	Sample treatment*
[28]	Rabilloud	500 mM disodium EDTA	Demineralize	10 volumes	Overnight	Combined with next step
		8 M Urea, 2 M thiourea, 1% CHAPS, 50 mM dithiothreitol	Extract protein	2×10 volumes	48 hr then 24 hr	D/L or precip
[30]	C+C	2% SDS in 500 mM disodium EDTA	Demineralize and extract protein	2×10 volumes	48 hr then 24 hr	D/L or precip
[15]	Embery 1	10% disodium EDTA	Demineralize	10 volumes	7 days	D/L
	Embery 2	4 M GuHCl in 50 mM sodium acetate (pH 5.8)	Extract protein	10 volumes	72 hr	D/L
[20], [21]		500 mM disodium EDTA	Demineralize	4 volumes	Overnight	Discarded
	Schweitzer 1	500 mM disodium EDTA	Demineralize	2×2 volumes	72 hr	Combined with next step
		6 M GuHCL in 100 mM Tris pH 7.4	Extract protein	2 volumes	Overnight at 60°C	D/L or precip
	Schweitzer 2	6 M GuHCL in 100 mM Tris pH 7.4	Extract protein	2×2 volumes	48 hr then overnight at 60°C	D/L or precip

*Dialysis and lyophilization is abbreviated D/L. Precip represents chloroform∶methanol∶water precipitation performed on half of the supernatant. Volumes correspond to the number of milliliters of buffer multiplied by the grams of bone powder.

**Table 4:** Summary of Jiang method and Buckley method.

Citation	Sample Name	Solution	Purpose	Volume	Incubation Time	Sample treatment*	Additional notes
[13]	Jiang 1	1.2 M HCl	Demineralize	6.5 mL	Overnight	D/L	
	Jiang 2	6 M GuHCL in 100 mM Tris pH 7.4	Extract protein	6.5 mL	72 hr	D/L	
	Jiang 3	500 mM tetrasodium EDTA in 6 M GuHCl in 100 mM Tris pH 7.4	Demineralize and extract protein	6.5 mL	72 hr	D/L	
	Jiang 4	6 M HCl	Extract protein	6.5 mL	Overnight	D/L	
[10], [11]	Buckley 1	0.6 M HCl	Demineralize	10 volumes	4 hr	D/L	
	Buckley 2	50 mM NH_4_HCO_3_	Extract protein	24.7 mL	5 hr at 65°C	D/L	Pellet neutralized with water before addition of ammonium bicarbonate

*Dialysis and lyophilization is abbreviated D/L. Volumes correspond to the number of milliliters of buffer multiplied by the grams of bone powder.

**Table 5:** Yields from ∼1.3 g of bone powder of each extraction protocol described in Table 1.

Lyophilized Sample	Total mass (mg)	%Yield	Lyophilized Sample	Total mass (mg)	%Yield
FH2 6 M 50 mM	385.3	29.5	MOD FH 50 Total	385.3	29.5
FH2	361.8	27.8	Jiang Total	372.5	28.7
Wendel 2	352.5	27.3	FH Total	361.8	27.8
Jiang 4	354	27.3	Wendel Total	356.5	27.6
Rabilloud	222.1	17.0	MOD FH 100 Total	220.3	16.9
FH2 6 M 100 mM	220.3	16.9	Embery Total	56.3	4.3
C+C	124.3	9.8	Schweitzer Total	20	1.6
Embery 1	41.5	3.2	Buckley Total	20.3	1.6
Jiang 3	15.9	1.2	Gurley Total	15.6	0.0
Embery 2	14.8	1.1			
Buckley 1	10.9	0.8	Moa Buckley 1	18.9	2.1
Schweitzer 2	10.3	0.8	Moa Buckley 2	35.6	3.9
Schweitzer 1	9.7	0.8	Moa FH1	5.4	0.6
Buckley 1	9.4	0.7	Moa FH2	186.6	20.7
Wendel 1	4	0.3			
Jiang 1	2.6	0.2	Moa Buckley Total	54.5	6.0
Gurley 2	14.7	0.0	Moa FH Total	192	21.3
Gurley 1	0.9	0.0			
FH1 6 M 100 mM	B.D.	B.D.	Precipitated Samples	Total mass (mg)	%Yield
FH1	B.D.	B.D.	Wendel 2	3188.6	246.8
FH1 6 M 50 mM	B.D.	B.D.	Schweitzer 1	1293.2	100.3
Jiang 2	B.D.	B.D.	C+C	731	57.5
			FH2	642.2	49.3
			Rabilloud Precip	569.6	43.6
			Schweitzer 2	70.1	5.4

B.D. refers to below the limit of detection for the balance used (0.1 mg). Total values correspond to lyophilized samples only and are calculated by addition of each step of an individual protocol.

**Table 6:** Ostrich enzyme-linked immunosorbent assay results showing means plus or minus one standard deviation.

	Collagen	Osteocalcin	Hemoglobin
Buckley 1	0.29±0.02 (+)	0.05±0.07 (+*)	0.24±0.02 (++)
Buckley 2	0.12±0.02 (+)	0.00±0.013 (*)	−0.01±0.01 (−)
C+C	0.03±0.01 (−)	−0.03±0.01 (*)	0.19±0.03 (++)
Embery 1	0.04±0.00 (−)	−0.03±0.00 (*)	0.06±0.00 (+)
Embery 2	0.02±0.35 (−)	−0.01±0.03 (−)	−0.01±0.00 (−)
FH 1	3.25±0.01 (+++)	0.04±0.03 (−)	0.19±0.01 (++)
FH 2	0.889±0.02 (+++)	0.07±0.01 (+*)	0.85±0.03 (+++)
FH1 6 M 100 mM	0.95±0.02 (+++)	0.02±0.01 (−)	0.14±0.00 (+)
FH2 6 M 100 mM	0.13±0.03 (+)	−0.02±0.01 (*)	0.07±0.02 (+)
FH1 6 M 50 mM	1.64±0.08 (+++)	0.00±0.01 (−)	0.28±0.05 (++)
FH2 6 M 50 mM	0.23±0.26 (+)	−0.04±0.01 (*)	0.05±0.01 (+)
Gurley 1	0.05±0.04 (−)	−0.030±0.00 (−)	−0.04±0.00 (−)
Gurley 2	3.02±0.15 (+++)	0.03±0.01 (−)	0.24±0.08 (++)
Jiang 1	0.50±0.09 (++)	0.04±0.03 (*)	0.18±0.02 (++)
Jiang 2	0.60±0.01 (++)	0.00±0.00 (*)	0.20±0.01 (++)
Jiang 3	0.42±0.05 (+)	−0.03±0.01 (*)	0.18±0.06 (++)
Jiang 4	0.23±0.03 (+)	−0.08±0.02 (−)	0.12±0.04 (+)
Rabilloud	0.11±0.01 (+)	0.00±0.01 (*)	0.05±0.00 (+)
Schweitzer 1	2.30±0.07 (+++)	−0.08±0.02 (−)	0.90±0.03 (+++)
Schweitzer 2	1.72±0.11 (+++)	−0.05±0.02 (−)	0.43±0.03 (+++)
Wendel 1	2.32±0.27 (+++)	−0.03±0.00 (−)	0.214±0.09 (++)
Wendel 2	0.88±0.07 (+++)	−0.03±0.00 (−)	0.53±0.06 (+++)

Values correspond to absorbance at 405 nm. − represents no detected absorption. + represents between two and ten times the average absorbance of buffer controls, ++ represents 10 and 20 times, and +++ represents >20 times.

*Represents bands on gels consistent in molecular weight with osteocalcin.

We also compared the yield of precipitated samples to that of dialyzed samples and found that precipitation gives anomalously high yield values. For example, Wendel 2 and Schweitzer 1 methods produce greater yield than the original bone mass. This implies that the precipitation method causes precipitation of a large amount of salt, in addition to the proteins of interest. The tested precipitation method is designed for low concentration salt or detergents, so it may not be optimal for these extraction types. Acetone precipitation was not used because we found that many of the tested solutions precipitate, even though it has been used on bone extractions previously [13]. The current data suggest that dialysis is a better method of desalting for these highly concentrated salt solutions; although, ethanol precipitation of guanidine HCl solutions [62] may be a more effective means of protein concentration and purification than either the chloroform-methanol-water precipitation or dialysis used here.

All tested protocols on ostrich bone, with the exception of the Gurley 1 method, show silver staining on SDS-PAGE gels (Fig. 1). All of these methods have been found to solubilize proteins to varying degrees; however, some methods provide better apparent quality on gels. For example, intense bands are visible for the Buckley 1,2 and Jiang 1,2 methods whereas the Schweitzer 1 and Embery 2 methods show smears for ostrich extractions. The cause of the smearing has not been investigated here; however, smearing may relate to residual EDTA left after dialysis because methods using HCl as a demineralizing agent show distinct, intense bands (Buckley 1, Jiang 1:Fig. 1). This residual EDTA may be the reason for the largest yield values (i.e., the greatest yield occurred for extractions utilizing EDTA [Table 5]; whereas, methods utilizing HCl typically have low yield amounts). Residual EDTA is not unexpected and has been shown to require ∼15 washes to completely remove it [14]. Alternatively, the smearing may indicate that the HCl is hydrolyzing small protein fragments leading them to electrophorese off of the gel, resulting in intense bands indicating a more pure extraction of intact proteins. This alternative, however, is unlikely because smears occur across the entire molecular weight range in Schweitzer 1, and bands are present on other methods indicating EDTA is most likely interfering with electrophoresis. The most intense bands occur on procedures that do not utilize EDTA or EDTA is a minor component in demineralization.

The method of solubilization is important depending on the protein of interest. Almost all of the extractions tested, with the exception of the SDS extraction, showed detection, based on light absorbance, of binding for anti-collagen I antibodies (Table 6). High molecular weight bands or smears are visible on gels for guanidine HCl, urea/thiourea, and ammonium bicarbonate extractions supporting the ELISA data (Fig. 1). Antibody binding for anti-hemoglobin was also detected for many extractions, including the SDS extraction, but was not detected for the ammonium bicarbonate extraction following HCl demineralization (Table 6). This is despite being observed in all subsequent Jiang extractions (guanidine HCl, EDTA/guanidine HCl, and HCl). This result was unexpected and may indicate that ammonium bicarbonate is not a good buffer for extraction of hemoglobin from bone and a different denaturing buffer may be required to collect this molecule. Very few extractions showed binding for antibodies to osteocalcin (Table 6) for ostrich; however, apparent osteocalcin bands are present in many different extraction types on gel (Fig. 1, Table 6). The difference between the ELISA and gel data may relate to the amount of serial dilution required for ELISA that is not necessary for gels, and/or the difference in solubility of osteocalcin in the dilution buffers between the two assays. In addition to solubility, osteocalcin may not adhere to the ELISA plates or is prevented from sticking to the plates by the abundant collagen in the samples preventing detection by antibody. The data suggest that the best methods for osteocalcin extraction, like collagen extraction, are ones utilizing HCl for removing mineral instead of EDTA. Given the suggested relationship between osteocalcin and collagen I [64], the greatest dissociation of osteocalcin and acid-insoluble collagen I should occur at low pH, in the presence of aqueous phosphate and calcium, as would be expected to occur during demineralization of bone.

The best protein extraction methods appear to be those that follow an HCl demineralization step, which induces ‘swelling’ of the collagen matrix [65] and increases the ability of both collagen I and collagen-associated proteins to go into solution. The use of 0.6 M HCl in the Buckley method [10], [11] is optimized for reduction of acid-induced hydrolysis for archaeological bone [65] and retention of acid-insoluble collagen I in the pellet for subsequent extraction. EDTA is a less optimal method for demineralization because it is slow [65] and requires a greater number of washing steps (∼15 washes; [14]) to fully desalt.

Based on the apparent quality of ostrich bone extractions, the methods in [10], [11] utilizing HCl for demineralization and [16], [17] utilizing EDTA for demineralization were performed on the moa bone. The methods in [10], [11] were chosen because they use less harsh conditions (i.e., lower concentration HCl) for demineralization than [13]. The methods used in [16], [17] also allow for investigation of exogenous protein because they utilize a pre-demineralization extraction step that could be beneficial for taphonomic studies of archaeological and paleontological bone. These two methods were chosen because one appears to be very clean [11] and the other has close to the highest yield value [16], [17], although it likely contains residual salt. These two methods also represent each of the common demineralization protocols tested throughout.

Even though, we tested these methods on moa bone, it was not our intent to demonstrate endogeneity or to make claims for advancing our understanding of moa biology or evolution. We used this bone to show that these methods can also be applied with equal efficiency to fossil and subfossil bone. For all studies employing molecules recovered from ancient bone to determine evolutionary relationships, rates, or direction, environmental controls, consisting of at least depositional sediments and laboratory buffers, treated in tandem with test samples, is critical for evaluation of potential contamination in the extractions.

### Ancient Bone Protein Extraction Methods

**Figure 2:**
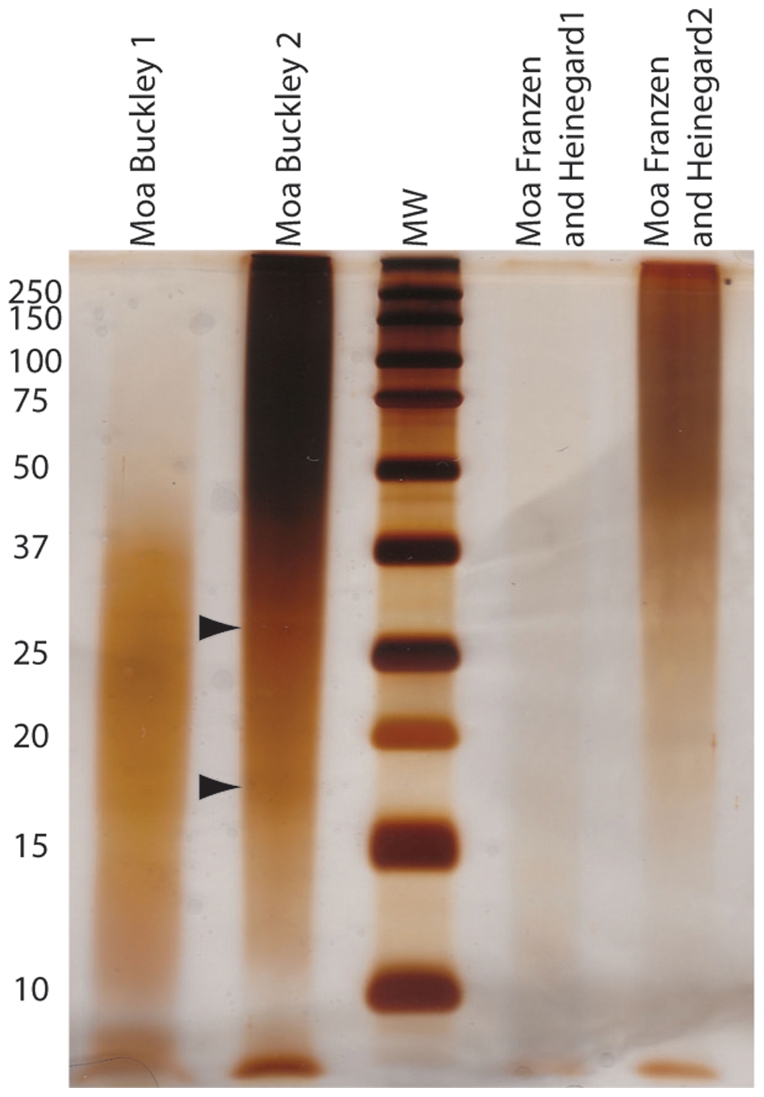
SDS-PAGE gel of moa extractions. Arrowheads indicate faint bands visible through the smearing.

Efficient extraction of ancient proteins is imperative to their study; especially, if only a small amount of original protein remains in the bones. The two extractions performed on the moa bone follow a similar pattern to the ostrich extractions, except that the resultant powders are a light brown color. This coloration may correspond to the appearance of the bone pre-extraction or other diagenetic products that co-extract (e.g., humics), but additional characterization is needed to determine the color's origin. Of the two methods tested, the Franzén and Heinegård method yields a greater amount of material than the Buckley method (Table 5), yet this may be a product of sample purity as observed in the ostrich extractions. Antibodies to collagen I show detectable binding, as measured by absorbance, in three of the four extraction parts (Table 7). Smearing at high molecular weights (Fig. 2) is visible for these three as well, consistent with other studies of ancient material [20], [21], [66]. Unexpectedly, the Buckley 1 method does not show high molecular weight silver binding, but instead only has binding below 50 kDa (Fig. 2) supporting that it is appropriate for demineralization with minimal extraction of high molecular weight species for this previously unprocessed/discarded step. The solubilization of proteins in HCl without high molecular weight species, like collagen, may allow for characterization of NCPs from ancient bone. In this case, the extraction method (Buckley) yields a better signal than the FH method for all proteins assayed. Other analytical techniques (e.g., mass spectrometry) are required to fully characterize these samples and determine their protein content. Because mass spectrometry gives primary sequence information, it makes molecular data obtained from ancient bone more useful. The primary sequence allows for formation of molecular phylogenies, comparisons of molecular evolution in deep time, and determination of endogeneity. The addition of molecular information to morphological phylogenies may help elucidate relationships that otherwise would not be resolved. Determination of endogeneity is extremely important for ancient bone because, without sequence information, all molecular information could be considered contamination.

**Table 7:** Moa enzyme-linked immunosorbent assay results showing means plus or minus one standard deviation.

	Collagen I	Hemoglobin	Osteocalcin
Moa Buckley 1	0.35±0.25 (−)	−0.15±0.071 (−)	0.38±0.43 (−)
Moa Buckley 2	2.34±0.02 (+)	0.48±0.01 (+)	1.37±0.152 (+)
Moa FH1	2.04±0.12 (+)	0.11±0.06 (−)	0.71±0.10 (+)
Moa FH2	1.542±0.15 (+)	0.19±0.08 (−)	0.05±0.58 (−)

Values correspond to absorbance at 405 nm. − represents no detected absorption. + represents at least two times the average absorbance of buffer control.

### Conclusions

Collecting the protein content in bone follows a very standard pattern of demineralization and solubilization independent of assay type. We have shown that all of the tested methods for solubilization, including those typically used on non-biomineralized tissues, can work for bone proteins. The choice of buffers used to extract proteins will ultimately depend on the protein of interest and/or analytical application. Protocols utilizing HCl for demineralization result in some of the purest extractions, but may result in unwanted hydrolysis, whereas those utilizing EDTA usually leave residual salt and therefore require an additional purification step. Both ammonium bicarbonate and guanidine HCl extract bone proteins well and either is suitable for many types of analyses. Ammonium bicarbonate extractions require less desalting, and therefore less sample loss and fewer opportunities for contamination. This extraction type is more appropriate for mass spectrometry than guanidine HCl making it useful for characterization of ancient samples with little remaining protein.

The literature contains many variations on extraction protocols for use in recovering protein from bone. However, it is necessary to modify these to increase yield, concentration, and purity while decreasing artifact or contamination opportunities, particularly when working with ancient samples. Some protocols have been optimized to increase yields for a particular protein (e.g., collagen I; [10], [11]), but still result in low total yields. The purest samples only result in 1–2% yield, which is only 5–10% of the total bone protein (assuming ∼20% of the total bone mass is protein; [63]). Moderate increases in protein recovery may allow for greater characterization of non-collagenous proteins from both extant and ancient bone and will allow for a greater understanding of protein preservation into deep time.

## Materials and Methods

### Bone Samples

Ostrich cortical bone fragments were degreased using a 10% Zout solution (Dial Corporation) to more closely approximate ancient bone. They were then frozen in liquid nitrogen and powdered using mortar and pestle. Very dark brown to black cortical bone fragments from an 800–1000 yr old moa (MOR OFT255, courtesy J. Horner) were powdered using mortar and pestle. MOR OFT255 has been briefly described as originating from cave deposits in New Zealand [67].

### Bone Extractions

All extractions were performed at room temperature on ∼1.3 g of bone, and solutions were centrifuged at 6000 rcf for 15 minutes between each step and supernatants were collected, unless otherwise noted (see Tables 1, 2, 3, 4). After collection of the supernatants, each extraction was dialyzed for 4 days against e-pure water in a 2000 MWCO Pierce Slide-A-Lyzer Dialysis Cassette to remove salt and lyophilized to completion. Because some evidence suggests that degraded organic material may be adhering to dialysis membranes, we compared recovery rates between dialysis and protein precipitation. Half of the supernatant from [16], [17], [20], [21], [24], [25], [28], [30] were dialyzed while the second half was precipitated using a chloroform∶methanol∶water precipitation method [68]. The yield for each desalting protocol was kept separate in Table 5. The resultant lyophilates were weighed, and yield was calculated by dividing the mass in milligrams of lyophilate by the original mass of bone powder and multiplying by 100 according to equation 1.
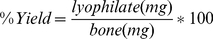
(1)The total yield of individual procedures was calculated by adding the yield of each step. For brevity, all extractions are described in Tables 1, 2, 3, and 4. Yield values in Table 5 are given for each step of each protocol. Multipart protocols are added subsequently to give a total yield value.

### Extraction of Moa

Approximately 0.9 g of powder were aliquoted and, based upon the results from extant bone samples, extracted using the [10], [11], [16], [17] methods. The HCl supernatant from [10], [11] and supernatants from [16], [17] were dialyzed against water and lyophilized as described above; the ammonium bicarbonate supernatant [10], [11] was dried using a speed vacuum. Yield was calculated following equation 1.

### Enzyme linked immunosorbent assay (ELISA)

Each ostrich extract was resuspended in 1× phosphate buffered saline (PBS) to a final concentration of 1 µg/µl. Because it was expected that some moa proteins would be degraded, each moa extract was resuspended to a final concentration of 10 µg/µl. Fifty microliters of each extract and PBS blanks were aliquoted to Immulon 2HB UBottom (Thermo Scientific) 96-well ELISA plates and allowed to incubate for four hours at room temperature. Wells and plated antigen were incubated for four hours at room temperature or overnight at 4°C with an antibody dilution buffer. This buffer consisted of 5% bovine serum albumin (BSA) in 1× PBS solution with Tween 20 and Thimersol. It was used to saturate the well with protein to inhibit spurious binding of primary or secondary antibodies and reduce background signal. This buffer was removed, and 200 µl of primary antibodies (polyclonal anti-ostrich hemoglobin [custom antibodies produced by Genscript], polyclonal anti-chicken collagen I [United States Biological], diluted 1∶400 in the above antibody dilution buffer, and monoclonal anti-osteocalcin [Abcam] diluted 1∶100) were allowed to incubate with plated antigen for four hours at room temperature or overnight at 4°C. Primary antibodies were removed and wells were washed 10 times in an ELISA wash buffer, consisting of 1× PBS with 0.1% Tween 20. After washing, each well was incubated in 100 µl of secondary antibody (alkaline phosphatase conjugated goat anti-rabbit IgG [ZYMED], diluted 1∶2000 in dilution buffer) for two hours, then washed 10 additional times. Antibody-antigen complexes were detected using a p-nitrophenylphosphate tablet (Sigma) diluted in a substrate buffer consisting of 9.8 mM diethanolamine and 10.5 M MgCl_2_. Positive binding was quantified at 405 nm using a Molecular Devices THERMOmax microplate reader for ostrich samples and a Molecular Devices Spectra Max Plus for moa samples. Data were acquired in Softmax Pro 4.8.

### Gel Electrophoresis

Lyophilates resulting from the above extractions listed in tables 1, 2, 3, and 4 were resuspended in 1× Laemmli buffer (Bio-Rad) with 20 mM DTT (Hoefer) to a stock concentration of 80 µg/µl. 3 µl of each sample was diluted in an additional 27 µl of 1× Laemmli buffer (final concentration 8 µg/µl) and 5 µl was added to the wells of 15% sodium dodecyl sulfate polyacrylamide gels (SDS-PAGE). Samples were electrophoresed at a constant voltage of 240 V for 1 hour to separate components by size and/or charge. Silver staining was performed at room temperature following [69].
